# Perfect Information vs Random Investigation: Safety Guidelines for a Consumer in the Jungle of Product Differentiation

**DOI:** 10.1371/journal.pone.0146389

**Published:** 2016-01-19

**Authors:** Alessio Emanuele Biondo, Alfio Giarlotta, Alessandro Pluchino, Andrea Rapisarda

**Affiliations:** 1 Department of Economics and Business, University of Catania, Corso Italia 55, 95129 Catania, Italy; 2 Department of Physics and Astronomy, University of Catania, Via S. Sofia 64, 95123 Catania, Italy; 3 INFN Section of Catania, Via S. Sofia 64, 95123 Catania, Italy; Universitat Jaume I, SPAIN

## Abstract

We present a graph-theoretic model of consumer choice, where final decisions are shown to be influenced by information and knowledge, in the form of individual awareness, discriminating ability, and perception of market structure. Building upon the distance-based Hotelling’s differentiation idea, we describe the behavioral experience of several prototypes of consumers, who walk a hypothetical cognitive path in an attempt to maximize their satisfaction. Our simulations show that even consumers endowed with a small amount of information and knowledge may reach a very high level of utility. On the other hand, complete ignorance negatively affects the whole consumption process. In addition, rather unexpectedly, a random walk on the graph reveals to be a winning strategy, below a minimal threshold of information and knowledge.

## Introduction

Since the seminal work of Chamberlin [1], the economics literature on monopolistically competitive markets has been rooted on the idea of product differentiation [2]: goods are provided by many producers in several versions and models, despite they aim at satisfying the very same need. Such a market framework can be easily experienced everyday in real-life markets, where several products compete with each other in offering very similar services to the final consumer.

Product differentiation has been studied from two perspectives: (i) *horizontal*, often referred to as spatial differentiation [3, 4], and (ii) *vertical*[5–8]. If a good is defined as a bundle of characteristics (in the sense of Lancaster [9]), the distinction between horizontal and vertical differentiation is that the former refers to goods with different features and the same qualitative level, whereas the latter takes into account differences in quality. However, as Cremer and Thisse have shown [10], results of Hotelling-type models and vertical differentiation models are basically equivalent. In this paper we assume that the differentiation has a comprehensive characterization: in fact, different features and qualitative levels will be described in a unique multidimensional environment. In other words, we refer to a higher-order differentiation concept, which embeds both the number of product features and their intensity. More precisely, we implicitly take a multi-criteria approach, in which every feature (including price) is suitably modeled and weighted: the reader can refer to literature about the *multi-attribute utility theory* and the *outranking* approach, [11][12].

Consumer’s behavioral choice is studied in the literature from several, yet complementary, points of view. From a buyer’s perspective, differentiation among goods is advantageous insofar as it helps in seeking a feasible good offering the highest satisfaction. In this sense, an increase in differentiation is usually associated to a positive value for buyers, because it increases the possibility of a making a selection that is close to an ideal target. For a detailed analysis of the possible reasons why consumers seek variety, we refer the reader to [13]. Monopolistic competition and oligopoly characterize the largest part of the real market experience. These two market structures share many common characteristics; however, they differ from each other in the way the total market output is looked at. In fact, oligopolistic firms explicitly consider the effect of their role in determining the total market output, whereas monopolistically competitive firms consider the aggregate market output as exogenously given in the process of setting their individual production, [14]. We focus our attention on the demand side of a *monopolistically competitive market*, where firms’ supply composition is taken as an exogenous environmental configuration. Our goal is to study a consumer’s individual choice process, that is, to analyze the way in which she selects a final product among several version of it supplied by different brands. To this aim, we make the (non-simplifying) assumption that the consumer considers quality, features, and prices as a unified multi-criteria discrimination concept.

Market failure due to asymmetric information occurs when the buyer and the seller have two different informative sets and the more aware of them tries to extract a supplementary benefit at the expenses of the other [15–17]. Indeed, the case of a consumer who does not know the complete characterization of the goods she needs must be considered: that consumer chooses blinded by her ignorance [18]. The result is a lower level of satisfaction than the potential one. This may require a policy intervention to solve the resulting inefficiency. To further support this point of view, consider the perverse effect of differentiation: the “tyranny of choice”—a term coined by Schwartz [19]—represents the situation when the abundance of available choices on the market may even become undesirable if the consumer cannot count on a valid consciousness to guide her selection. In this respect, some authors stress the role of advertisement in informing consumers [20]. However, adverts may barely provide a neutral source of knowledge. Thus, the model here proposed will show effects of advertising.

The relevance of *information* and *knowledge* in consumption is widely recognized, because of their ability to influence the consumer’s attitude in perception and searching. Indeed, some analysis supports the point of view that a lack of knowledge may truly generate a reduction of the consumer’s judgement capacity [21]. In our opinion this conclusion cannot be fully ascribed to the well-known “bounded rationality” concept, which appears more linked to the completeness of the informative set, as advocated in [22, 23] and in [24]. In this paper we explicitly emphasize the semantic difference between the two concepts of “information” and “knowledge”. Our interpretation of these two terms relies on a suggested distinction between (i) what the consumer knows with regards to the current transaction being concluded (which should be considered as “information”, as generally accepted in literature), and (ii) what the consumer knows in a broader sense, in the form of cultural background and capability to understand and to evaluate (which is a notion of “knowledge”). The interested reader may refer to existing surveys [25, 26]. A possible rationale for the reduction of the consumer’s judgement capacity can be ascribed to the complexity of choice environment [27]. In fact, it is known that consumers decide to make purchases for many reasons, which range from very basic needs to utmost volatile instincts. As a consequence, even their perceived desires/needs and the way they are expressed respond to a very wide set of stimuli, as documented in an ample literature [28–30]. Also, there is evidence that choices made in consumption may affect the development dynamics of the economy [31].

We approach the problem of individual consumer’s choice by describing the different phases of the informative process that leads the buyer to her final selection. In this sense, we assume that the consumer is able to build an “experiential cognitive map”, where all the variables are taken into account in a spatial sense. Our approach basically belongs to the well-known framework of consumer decision-making research [32], which considers consumption as a process that generally involves the following phases: (1) problem recognition (i.e., market selection according to the need being satiated); (2) information search; (3) evaluation of alternatives; (4) final choice (i.e., purchase); (5) post-purchase evaluation. We assume that the consumer has chosen the market where to enter, and focus on phases (2), (3), and (4). We will neglect phase (5).

In order to build a spatial configuration, here we use a Hotelling-type approach [3, 4], to qualify the satisfaction in terms of the distance from the “perfect choice”. Our model informally follows a multi-attribute approach: we assume that the consumer’s exploration will reveal her behavioral attitude in recognizing and appreciating desired characteristics. Thus, the set of available options is analyzed, and, after comparisons, the most satisfying alternative is chosen [33–35].

Market complexity affects many aspects of the decision process. In fact, motivational elements and topological configurations of attributes space unavoidably link the maximizing choice to the effort put in the search [36]. This is the reason why our model is based on two distinct features: (i) the consumer’s ability in discriminating similar goods, and (ii) her knowledge of the market structure. Furthermore, a very strong influence on present choices and decisions is exerted by similar previous experiences [37–41]. As a consequence, we assume that a purchase decision passes through an analysis that takes into account every cognitive element of the consumer’s activity, either present or stored in memory from past experiences [42]. The theoretical context employed here allows individuals to adaptively select strategies in order to manage the actual situation at their best [43][44].

In this paper, we investigate the existence of some “safety guidelines” to help consumers with different degrees of information to make their choices. Inspired by previous studies on the beneficial role of randomness in socio-economic systems, we actually test the effectiveness of several strategies to reach a final decision, ranging from the hypothetical scenario of perfect information, to a completely random walk. As it was found for financial markets [45–48], for career advancements in managerial organizations [49, 50], and for efficiency of political institutions [51], we anticipate that also in this case randomness gives positive results.

## The model: statics and dynamics

In this section we describe our model. We first deal with its static features, which are related to the exogenous market structure, the endogenous consumer’s characteristics, and the interaction between market and consumer. Successively, we describe the dynamical procedure employed by the consumer to explore the market, gathering information in an attempt to reach her target.

### The market

**Fig 1:**
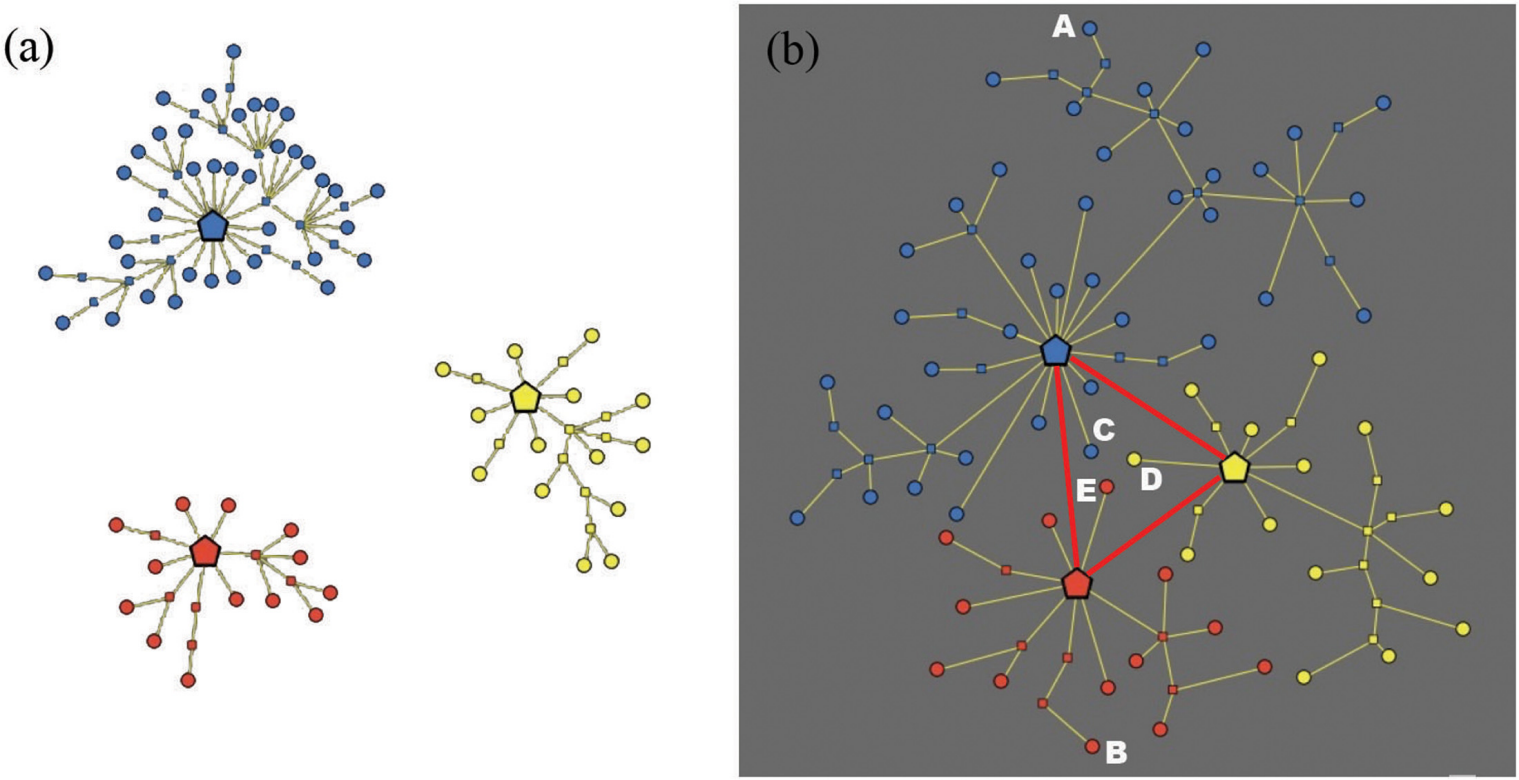
Graph representation of a market with three clusters. (a) Topological structure of the market. (b) Metric structure induced by consumer’s satisfaction. The clusters are identified by different colors. The red edges in the right picture represent the consumer’s knowledge (which is “complete” in this case).

The topological structure of the market is represented by a graph with three types of nodes, identified by different shapes, see Fig 1(a). Formally, the graph has the theoretic structure of a *forest*, that is, a free union of *trees* (connected acyclic graphs). Recall that a *graph* *G* is a pair (*X*, *E*), where *X* is a nonempty set of *nodes*, and *E* is a (possibly empty) set of (undirected) *edges*, with an edge being a subset of *X* having size two. Two distinct nodes *x*, *y* ∈ *X* are *adjacent* if {*x*, *y*} is an edge in *E*. A *path* in a graph *G* = (*X*, *E*) is a sequence (*x*_1_,…, *x*_*k*_) of *k* ≥ 2 adjacent nodes, in the sense that the set {*x*_*i*_, *x*_*i*+1_} is an edge in *E* for each *i* = 1,…, *k*−1. A graph is *connected* whenever any two distinct nodes are joined by a path, and is *acyclic* if there are no paths beginning and ending at the same node. For further details, refer to [52]. Each tree is star-shaped, and represents a “cluster”: for instance, the graph in Fig 1(a) has three clusters. The meaning of the three types of nodes is described below.

#### Central nodes

These nodes are located at the center of the trees composing the forest, and are denoted by pentagons. They are the *hubs* (i.e., highly connected nodes) of the graph, and may represent either a “brand” or a “category” of products. For example, in the market of cameras, we can look at each central node as a specific producer (Canon, Minolta, Nikon, etc.) or as a type of camera (bridge, mirrorless, reflex, etc.).

#### Terminal nodes

These are the *leaves* of the forest (i.e., the nodes with degree 1), and are denoted by circles. Recall that the *degree* of a node *x* is the number of nodes that are adjacent to *x*. The nodes adjacent to *x* are also called the *first neighbors* of *x*. The terminal nodes represent all final products that are sold on the specific market at hand. For instance, in dealing with the market of cellular phones, a terminal node may be a 4-tuple of the type 〈Apple, iPhone6, 32 GB, silver〉.

#### Intermediate nodes

These are the nodes that belong to paths connecting the hub of a cluster to the terminal nodes of the same cluster, and are denoted by squares. Intermediate nodes represent the distinct phases of a consumer’s *informative journey*, intended as a cognitive walk that guides her from a brand/category to a final product present in the market. To illustrate the role of intermediate nodes, consider the following example in a computer market. A consumer decides to buy a final product represented by the 6-tuple

$$\langle \text{Apple, Macbook Air, }13^{\prime \prime },\text{ i7 }-\text{ 2.2GHz, 8 GB SDRAM, 512 GB}\rangle .$$

Here the central node is the brand 〈Apple〉, the terminal node is a laptop with some specified features, and the intermediate nodes are all restrictions of the 6-tuple to the first *i* components. Specifically, the four intermediate nodes are 〈Apple, Macbook Air〉, 〈Apple, Macbook Air, 13′′〉, 〈Apple, Macbook Air, 13′′, i7—2.2 GHz〉, and 〈Apple, Macbook Air, 13′′, i7—2.2 GHz, 8 GB SDRAM〉. with *i* = 2,3,4,5. Thus, each product can be identified with an *n*-tuple of features specified by the sequence of *n*−1 informative steps needed to reach it, starting from the central node (which represents its first feature). In this way, a product actually sold in the market is completely determined by the topology of the cluster (brand/category) to which it belongs.

It is important to emphasize that in our approach the market structure is totally exogenous. Therefore, at this initial stage, the distinct clusters constituting the market are not connected to each other, and live in a space that is *not* endowed with any metric structure.

### The consumer

Once that both the number and the topological structure of the clusters are determined, the market has to be evaluated by consumers. In fact, each (type of) consumer has different features, preferences and perceptions, which constitute the “lens” through which the market is viewed by her. Below we describe in detail how the consumer’s point of view may affect the representation of the market, in terms of her: *satisfaction*, *knowledge*, and *discrimination ability*.

#### Satisfaction metric

The presence of a personal point of view naturally induces a deformation of the graph structure, which however leaves unchanged the original topology of the market. In fact, the market assumes a shape that reflects the existence of an underlying *satisfaction metric*, defined in an appropriate space and intrinsic to each (type of) consumer: see Fig 1(b), where, for the sake of graphical representation, we embed the graph into the two-dimensional space $\left(R^{2},d\right)$, with *d* denoting the standard Euclidean distance.

In order to better specify such an intrinsic satisfaction metric, we assume that each consumer has in mind an “ideal goal”, a hypothetical target product with several well-specified characteristics. This target, which may or may not exist in the market, occupies a specific position in the Euclidean space, say, $P^{*}\equiv \left(x_{P^{*}},y_{P^{*}}\right)\in R^{2}$. In particular, if the target is a real product sold in the market, then it is located on a terminal node of the graph.

A natural assumption of our model is that a consumer purchasing her ideal product P* will retrieve from it the maximum *ex-post* satisfaction, that is, Sat(P*) = 1. On the other hand, purchasing a product different from her target and corresponding to a terminal node $P\equiv \left(x_{P},y_{P}\right)\in R^{2}$ will provide her with a non-perfect satisfaction, that is, 0 ≤ Sat(*P*)<1 in this case. Note that the satisfaction Sat(*P*) retrieved from a non-ideal product *P* shall naturally depend on the Euclidean distance *d*(*P*,P*) = ((*x*_*P*_−*x*_P*_)^2^+(*y*_*P*_−*y*_P*_)^2^)^1/2^ of the chosen product *P* from the target P*. More generally, we define the satisfaction Sat(*P*) associated to any point $P\in R^{2}$ by

$$\text{Sat}\left(P\right)\;:=\;1\;-\;\frac{d(P,P^{*})}{d_{\max}}$$ (1)

where *d*_max_ is the maximum possible distance in the bounded part of the considered space $R^{2}$ (which is a square, in our case). Due to its generality, this definition of satisfaction works for any pair of nodes (not necessarily terminal).

It is important to note that in our model the distance between two terminal nodes is independent from the set of features of the corresponding products. For instance, the top blue node *A* and the lower red node *B* in Fig 1(b)—which are very far from each other in the underlying metric space—might well have very similar features; nevertheless, it is possible that the satisfaction deriving from buying *A* is very different from that deriving from buying *B*, even just because they belong to different clusters. Just to give a concrete example, think to a consumer whose target is an *Apple iPhone* with certain particular features: if she buys a *Samsung Galaxy* smartphone with very similar characteristics, then it is likely that she will be much less satisfied, since in this specific case the importance of the brand dominates the other features of the product. On the other hand, the three nodes of different colors placed at the center of the metric space (*C*, *D* and *E*) might present very different features despite being very close to each other (hence giving a similar satisfaction to the consumer).

#### Knowledge of clusters

In reality, consumers’ knowledge of the exogenous market structure may vary quite a lot. We measure knowledge in terms of perception of the existence of clusters; those clusters that are known to the consumer are labeled as *active*. Fig 1(b) graphically describes a deformed topological structure of a simple market with three clusters, as viewed by a perfectly informed consumer, i.e., a consumer with a maximum knowledge Kn_max_ = 1. In the represented case, the three clusters are all active, in fact their central nodes are connected to each other by red edges (whereas the *intra-clusters* links are colored in yellow). Note such a perfectly informed consumer could theoretically visit all the nodes of the graph during her informative journey, using both red and yellow edges.

**Fig 2:**
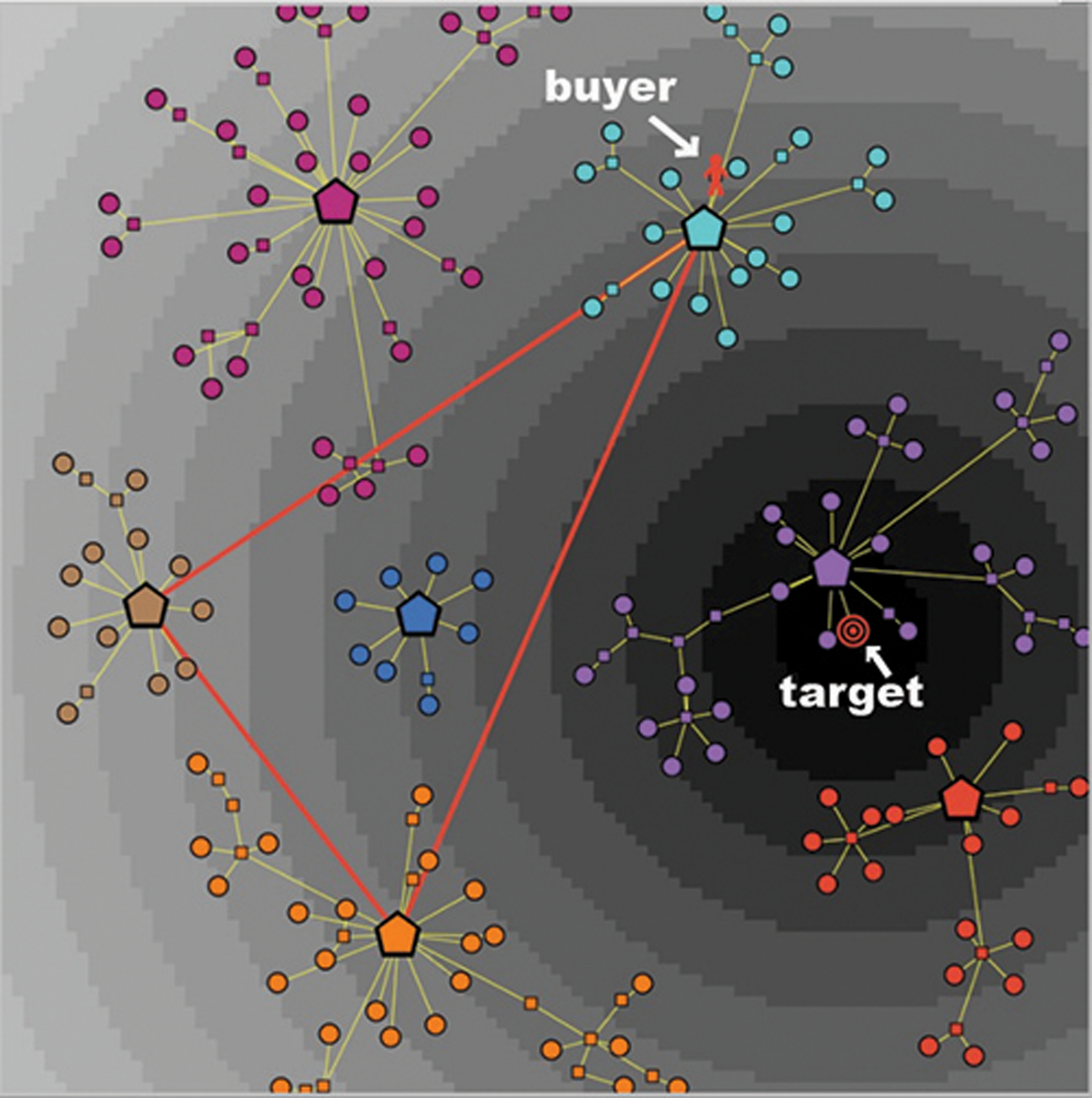
A market with seven clusters, as viewed by a consumer with moderate knowledge and high discrimination power. The consumer has a partial knowledge (Kn = 0.5) and a quite high number of indifference levels (*N*_Lev_ = 14), represented by annuli with different shades of gray. The indifference areas are centered at the target, which is located on a node, and is denoted by concentric red circles. The random initial position of the buyer is located on a node of the top-right cluster, and is denoted by a human shape.

The situation is quite different for a consumer with an imperfect knowledge Kn < 1. In Fig 2 we describe a market with seven clusters, as it is viewed by a consumer with a knowledge Kn = 0.5: in this case only three of the seven clusters are active, hence the informative journey of this type of consumer will be limited to the three corresponding brands/categories. In Fig 2 we also represent the following additional features:

(1) A possible position of the consumer at the beginning of her journey over the graph, indicated by a red human shape located on a node belonging to the top-right (active) cluster. In what follows, we refer to this initial node as the *source* of her psychological journey. (For example, in the process of searching for a new cellular phone to buy, the consumer might start her exploration from the node of the graph that represents—or is very close to—her old phone.)
(2) A possible position of her target product, indicated by a shape with concentric red circles. In the represented case, the target is located on a terminal node of the middle-right (inactive) cluster. (However, as already emphasized, the location of the target may well fail to coincide with a terminal node of a cluster, thus indicating that the consumer’s ideal product is not present in the market.)

In the described situation, despite the target coincides with a terminal node of the graph, the consumer will never be able to reach it, due to her imperfect knowledge: in fact, the target belongs to an inactive cluster. Therefore, the consumer’s greatest ambition can only be to get as close as possible to her target, while remaining within the subset of the market that is reachable by his partial knowledge.

#### Preference structure and discrimination ability

The last feature that influences the market evaluation by the consumer is her preference structure. In our model, we assume that the buyer has only a sort of “fuzzy” perception of the level of satisfaction associated to each point of the graph, until he actually reaches a node and physically explores it. Specifically, we distinguish (1) an “*ex-ante* perception”, which is the individual presumption of the satisfaction that a good can provide; and (2) an “*ex-post* evaluation”, which is the satisfaction that a good existing on the market actually provides. In this respect, the level of satisfaction associated to a given node of the graph represents what we call its “ex-ante utility”, which is just an estimate of its “effective utility” (measurable only after the consumer physically explore the node). We model this fuzzy preference structure by a *total preorder* on the set of nodes. Recall that a *total preorder* on a set *X* is a binary relation ≳ on *X*, which is *reflexive* (x≳x for each *x* ∈ *X*), *transitive* (x≳y and y≳z imply x≳z for each *x*,*y*, *z* ∈ *X*), and *complete* (x≳y or y≳x for each *x*, *y* ∈ *X* such that *x* ≠ *y*). In this case, the *indifference relation* ∼ associated to ≳, defined by *x* ∼ *y* if x≳y and y≳x, is an equivalence relation on *X*. This is a classical hypothesis in individual preference theory. Since the number of nodes is finite, the advantage of this framework is that it admits a *utility representation* with “thick” indifference classes. A binary relation ≳ on *X* is *representable* if there exists an *order-embedding* from *X* into the set R of real numbers, i.e., a map u:X→R such that for each *x*, *y* ∈ *X*, we have x≳y if and only if *u*(*x*)≥*u*(*y*). In this case, the function *u* is called a *utility representation* of ≳. It is well-known that a total preorder on a countable set (hence, in particular, on a finite set) is always representable: see, e.g., [53, 54] for a general discussion about utility representations and some technical results.

Fig 2 provides a graphical description of such a consumer’s preference structure. The $R^{2}$ space endowed with the satisfaction metric is partitioned into *N*_Lev_ equivalence classes, called *indifference levels* and represented by concentric annuli centered at the target. All the nodes in an annulus are *ex-ante* equally preferred by the consumer, i.e., they display the same *presumed* utility: Formally, if ≳ is the total preorder on *X* representing the preference structure of a consumer, ∼ is the indifference associated to ≳, and u:X→R is an utility representation of ≳, then we have *x* ∼ *y* if and only if *u*(*x*) = *u*(*y*). So each annulus can be seen as a fuzzy multidimensional representation of indifference curves. the further from the target an annulus is, the lower the presumed utility associated to the corresponding indifference level is, and vice versa. Clearly, a low value of *N*_Lev_ is typical of a consumer with a rather low ex-ante discrimination power, whereas a large value of *N*_Lev_ indicates a consumer with a high ability in discriminating among similar goods.

As a conclusive remark, note that we do not assume *a priori* the existence of a relationship between knowledge and discrimination ability. Therefore, we can identify several “categories of buyers”, which display various combinations of these two features. In the next subsection we illustrate how consumers interact with the market structure, and how this interaction—along with some additional individual features—determines different categories of consumers.

### Market-Consumer Interaction

Each central node (hub) of the graph can be considered as a “pole of attraction” for the consumer. In the case that hubs represent brands, the strength of such attraction depends on many factors, such as marketing suggestions, variety of offered products, advertising and promotional campaigns, communications and brand-management policies, etc. To simplify our analysis, in this paper we assume that the attractive power of a hub is only a function of the amount of products having that brand. In fact—in analogy with the gravitational field of a body in physics—we introduce the *mass* *M* of a hub, which is by definition equal to the number of leaves of the corresponding cluster. Note that the attraction effect generated by each hub becomes effective only if the consumer is aware of the presence of the corresponding cluster: said differently, “knowledge determines attraction”.

**Fig 3:**
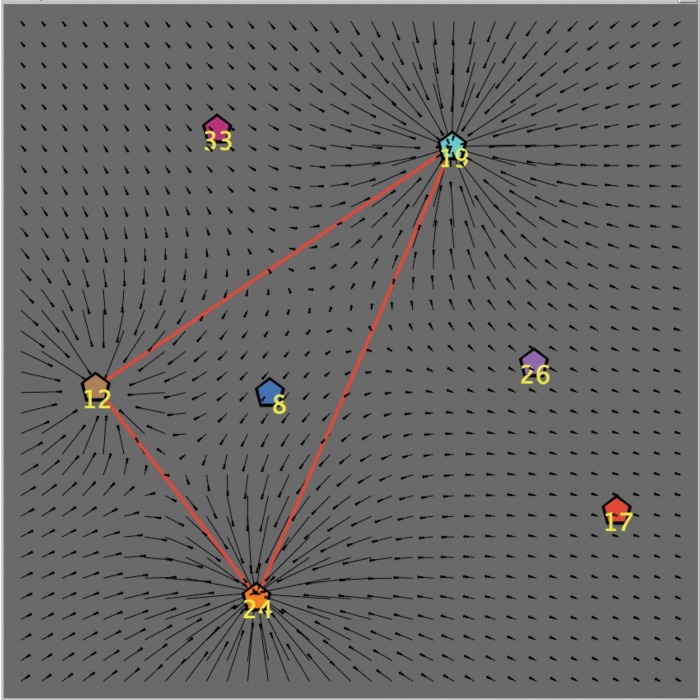
Market-consumer interaction: attraction field induced by three active hubs. The label next to each hub represent its mass, which is equal to the number of products (leaves) of the corresponding cluster. All intermediate and terminal nodes are not represented.

In Fig 3 we graphically represent the *attraction field* induced by the three active clusters of Fig 2 (the four inactive clusters have no effect whatsoever on the consumer, since she cannot even perceive their existence). For the sake of visualization, only the central nodes are reported in the figure. The integer number next to each hub is its mass *M*, which provides a discrete measure of the strength of directional attraction. The arrows of the attraction field may possibly push the consumer to choose in a certain direction. However, not every consumer will be affected in the same way by the field: in fact, as we shall argue later on, the more a consumer is well aware of her personal tastes/goals, the less she is influenced by market suggestions. In order to take into account this kind of behavior, next we introduce the last feature that characterizes a consumer in our model.

#### Awareness

This is the capability of the consumer to discern the features of a product without being subject to the influence of the market: the higher her awareness, the less likely that she makes her choice according to the market’s attraction field. In fact, a highly aware consumer will always take advantage of both her knowledge and her ex-ante discriminating power to effectively explore the market while moving towards the target. In our model we assume that awareness is a parameter Aw that varies in the closed interval [0,1].

Knowledge (Kn), discriminating ability (*N*_Lev_) and awareness (Aw) are the three parameters that allow us to distinguish among several categories of consumers, characterized by suitable combinations of these features. Here, for the sake of simplicity, we shall restrict the presentation of our results to a taxonomy of ten typical cases, presented in Table 1, in which the extreme values of the parameters better emphasize the differences among different types of buyers. In particular, consumers from type #1 to type #5 (with Kn = 0.5) display an imperfect knowledge of the market, while consumers from type #6 to type #10 (with Kn = 1) know the market perfectly. Within each of these two general categories, we have the extreme cases of consumers with either minimum (Aw = 0) or maximum (Aw = 1) awareness, as well as a special type of consumer who—as we shall describe later on—walks along her informative path completely *at random*. Finally, for non-random cases, we consider consumers with low and high discriminating ability (respectively, *N*_Lev_ = 4 and *N*_Lev_ = 20).

**Table 1:** Ten types of consumers characterized by different values of individual parameters. Only rather extreme values of knowledge (Kn), awareness (Aw) and discrimination ability (*N*_Lev_) are used. The special type of random consumer also appears.

Consumer	Kn	Aw	*N*_Lev_
#1	0.5	0	4
#2	0.5	0	20
#3	0.5	1	4
#4	0.5	1	20
#5	0.5	*random*	−
#6	1	0	4
#7	1	0	20
#8	1	1	4
#9	1	1	20
#10	1	*random*	−

These ten combinations of the main individual parameters describe a fictitious community made by several distinct (non-interacting) consumers, who aim at maximizing their own utility according to their subjective awareness, discrimination capacity and knowledge. As we explain in detail in the next section, each type of consumer will navigate the graph in search of her (existing or non-existing) target, following step by step (i.e., node by node) a personal informative path starting at the source (where she is located at time *t* = 0).

Next, we anticipate the possible outcomes of the informative journey of a generic consumer. Let *t*_*end*_ be the total number of explorative steps done by the consumer, who starts her journey at time *t*_0_. Further, let *P* be the product (present in the market) delivering the maximum satisfaction among the ones visited by her, and t* the step at which *P* is reached. (Thus, time is discrete, and t* is an integer between *t*_0_ and *t*_*end*_.) If Sat(*P*, t*) denotes such a maximum satisfaction, then the individual journey may give rise to four possible outcomes for the consumer, which depend on whether the target product exists (cases 1a and 1b) or not (cases 2a and 2b).

(1a) *The target coincides with a real node of the graph, and is actually reached*. In this case, the consumer ends her search at the target P* (i.e., *P* = P* and t* = *t*_*end*_), and, following Eq (1), her satisfaction is maximum (i.e., Sat(P*, t*) = Sat_max_ = 1).
(1b) *The target coincides with a real node of the graph, but is not reached*. In our model there are no constraints on the time that each buyer can spend while searching for her target. However—as we better explain in the next section—the consumer might eventually get trapped in an “informative cul-de-sac”, in which case she will buy the product corresponding to the maximum satisfaction reached until that moment. Specifically, if this maximum satisfaction is given by a certain product *P* reached at time t*, then the consumer’s satisfaction Sat(*P*, t*) is a number in the open interval (0,1).
(2a) *The target does not coincide with a real node of the graph, but the consumer reaches a node at minimum distance from it*. Whenever the ideal product does not exist in the market, choosing the “closest” product to it (at, say, time t*) is actually the best that the consumer can do. As a consequence, her satisfaction “should” theoretically be maximum, even if she will inevitable end her journey in a cul-de-sac. However, unless the consumer has a precise perception that the ideal product is not present in the market, at the end of her journey she will still believe that her original goal has not been fully accomplished. Therefore, as in case (1b), we have again 0 < Sat(*P*, t*)<1.
(2b) *The target does not coincide with a real node of the graph, and the consumer does not even reach a node that has the minimum distance from it*. In this case, there are no doubts that the consumer’s satisfaction is not maximum (in fact, lower than in the previous case). As in cases (1b) and (2a), her final satisfaction corresponds to the maximum one reached (at, say, time t*) during her exploration of the market, before being (inevitably) trapped in a cul-de-sac. Again, we have 0 < Sat(*P*, t*)<1.

Of course, the outcome effectively realized is also determined by several factors deriving from the heterogeneity of individuals, such as the effects of advertising, a lack of knowledge, a very inaccurate ex-ante preference structure, etc. Therefore, we expect that distinct types of consumers—endowed with different levels of knowledge, awareness and discriminating power—will behave quite differently. For instance, it is apparent that only consumers with Kn = 1 (from type #6 to type #10 in Table 1) will have the possibility to reach the target, provided that the latter is a real product on the market (outcome (1a)). On the other hand, a lack of connections between central nodes due to medium values of Kn (consumers from type #1 to type #5 in Table 1) will likely hinder the achievement of the target, even in cases in which the latter corresponds to a product existing in the market (outcome (1b)).

### Dynamics of the model

We now describe how a consumer effectively moves from a given source toward her target. This will enable us to evaluate differences among types of consumers in a statistically significant way.

To start, note that the fact that our consumer “has in mind” her target does not imply any knowledge about its exact location with respect to the nodes of graph. As a matter of fact, in the (rather unlikely) circumstance that the buyer possesses this perfect knowledge, she would immediately select an existing product that is as close as possible to the target, and there would be no explorative process. Instead, our model aims at describing the whole “consumption experience” from a dynamical perspective.

Actually, we assume that, as soon as the need of a certain good is perceived (here we do not explicitly address the question about the “repeated consumption” experience), the consumer has a (more or less) vague idea of what *would* satisfy her best. Thus, she starts her informative journey in order to find it, looking at the market structure according to her knowledge Kn, and inspecting existing goods in a way that is influenced by her awareness Aw and her discrimination ability *N*_Lev_. Specifically, the consumer starts at time *t* = 0 from a randomly chosen source node belonging to one of her active clusters, and moves at time *t* = 1 to another node of the graph, following either a yellow link (connecting nodes of the same cluster) or a red link (going from the hub of a cluster to another hub). At any time step *t* the consumer is on a certain node of the graph, and at time *t*+1 she moves to another node of the graph, following either a yellow link or a red link. To describe the algorithm that guides the consumer’s selection at each step of her journey on the graph, let us assume that at time *t* the consumer is on a given node *j* with degree *k*_*j*_. Now the question is: *how does the consumer select the neighbor node where to go at time t+1?*. In what follows, we give two distinct answers to this question, distinguishing between the two main categories of consumers: (1) non-random; (2) random.

**Fig 4:**
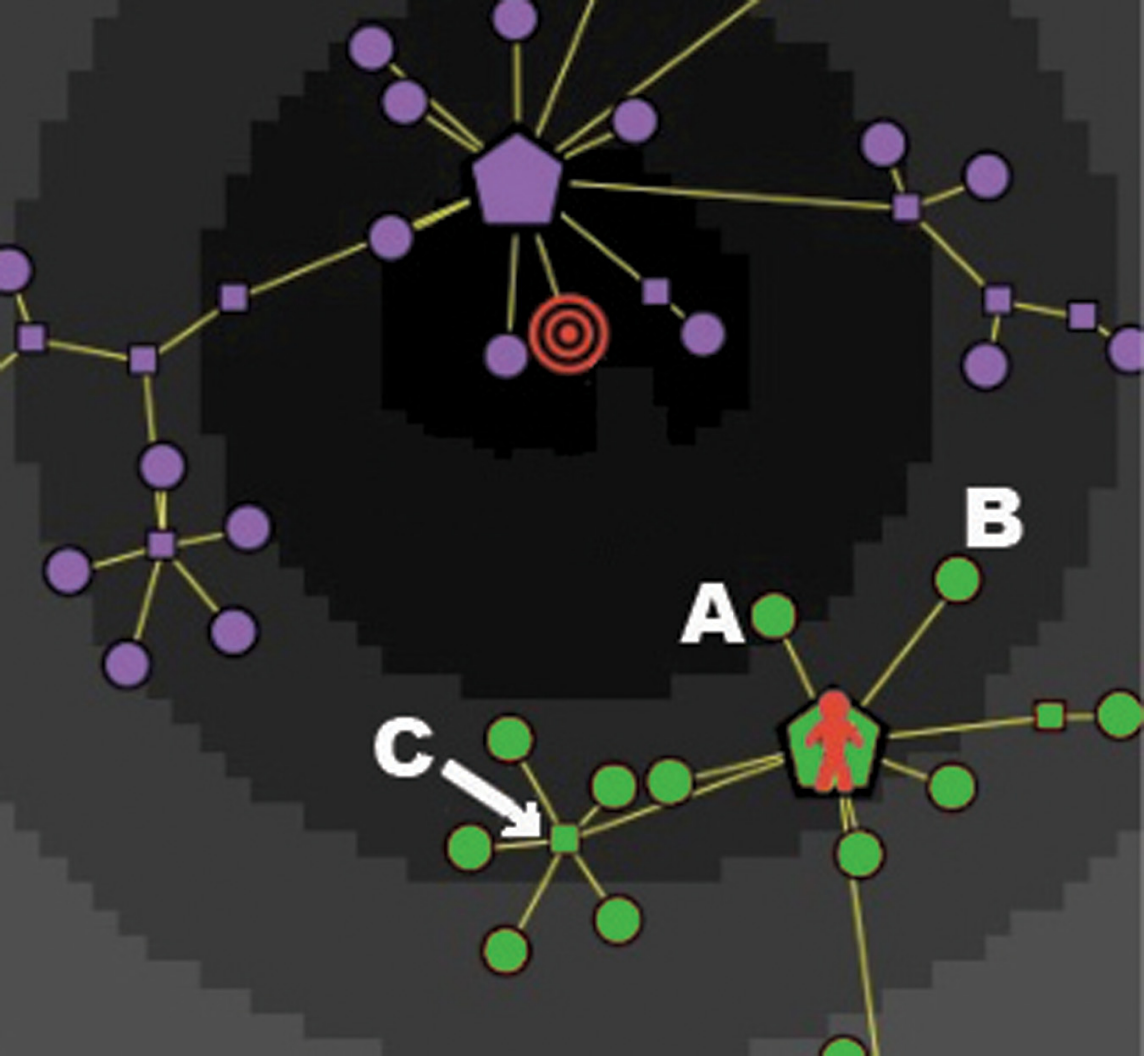
Dynamical rules determining the buyer’s next move: the case of high awareness. The consumer is well aware of the target, and tries to approach it by exploiting her high discrimination ability.

**Fig 5:**
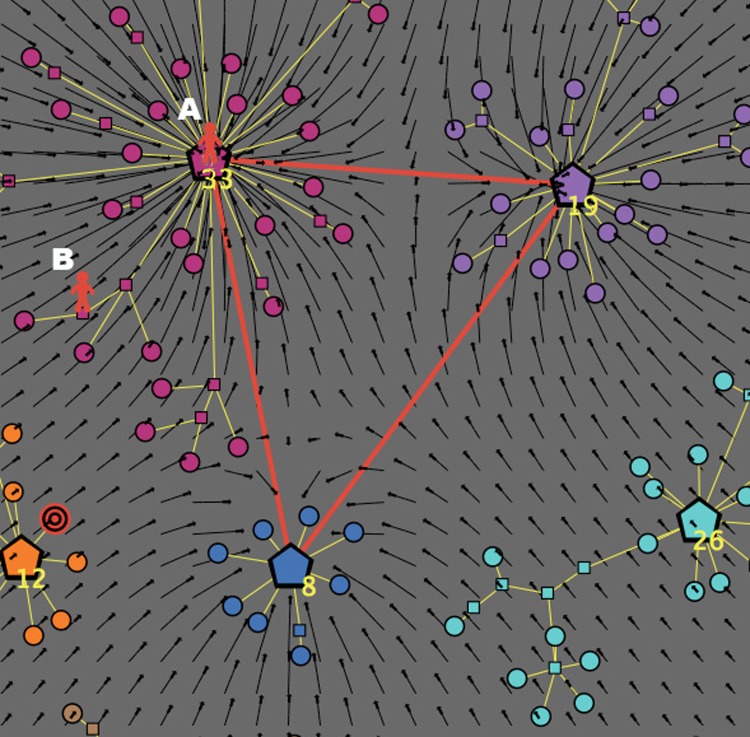
Dynamical rules determining the buyer’s next move: the case of low awareness. The consumer is highly influenced by the attraction field induced by her active clusters, without consideration for the target.

(1) For *non-random* consumers, the answer to the above question strictly depends on the values of the parameters Aw and *N*_Lev_. In fact, a consumer who is quite aware of her active segment of the market (i.e., of all the products belonging to her active clusters) will theoretically be guided only by her attempt to approach the target; on the other hand, the moves of a scarcely aware consumer will be largely influenced by the attraction field induced by her active clusters, almost independently of the position of the target. We translate these two opposite tendencies into, respectively, the “dynamical rules” (a) and (b) described below.
  (a) *With probability p = Aw, the consumer chooses one the first neighbors with highest degree among those with highest utility (according to her preference structure)*. More precisely, first the consumer ranks all “reachable” indifference levels by the total preorder that models her preference structure, that is, he determines the indifference level *L*_*i*_ containing a first neighbor that is the closest to the indifference level *L*_0_ of the target. If there is only one first neighbor in *L*_*i*_, then she chooses it. Otherwise, she chooses randomly one the first neighbors in *L*_*i*_ among those having maximum degree. In the majority of cases, there will be a unique first neighbor with maximum degree in the best reachable indifference class. The rationale guiding this selection process is very natural: the consumer selects according to a criterion of highest possible utility, and, in cases of *ex-equo*, she makes her choice according to the highest degree, since this selection increases her freedom of movement at the very next step of the informative process.
    In Fig 4 we represent the case of a highly aware consumer: at time *t*, she is on the hub of a cluster, located in an indifference class that is two levels below the best. According to our algorithm, at time *t*+1 the consumer will choose (with probability *p* = Aw) one of the first neighbors that belongs to the best reachable indifference class. In our case, there is only one node of this kind, namely *A*. Note that if node *A* were not to be present in the graph, then the consumer would have chosen node *C*, since this is the node with highest degree among the first neighbors in the best indifference level. For example, in Fig 4, the two nodes *C* and *B* belong to the same indifference class, but *C* (with degree 6) is better than *B* (with degree 1).
  (b) *With probability p = 1−Aw, the consumer select a first neighbor on the basis of the attraction field, without any consideration for the target*. In the example shown in Fig 5, a scarcely aware consumer is influenced by the attraction generated by the three active clusters (having masses equal to, respectively, 33, 19 and 8, as shown by the corresponding labels), whereas the target is located on another (unknown) cluster. In this case, the selection depends on the position *j* of the consumer at time *t*, according to the following two subcases:
    - if at time *t* the consumer is on a central node (position *A* in the figure), then at time *t*+1 she will move to one of her *k*_*j*_ first neighbors *with a probability proportional to the mass of each node* (for nodes that are not hubs, the mass simply corresponds to their degree); obviously, the central nodes of active clusters with a large number of leaves have a very high probability to attract the buyer (in our example, the central node with mass 19 will most likely be the buyer’s next choice);
    - if at time *t* the consumer is on an intermediate or a terminal node (position *B* in the figure), then at time *t*+1 he will move *at random* to one of her *k*_*j*_ first neighbors; in this way, the consumer has the opportunity to explore all the informative trees in her active clusters, without being forced to come back to the central nodes (which maximally attract her).
(2) On the other hand, a consumer may decide to walk along her informative path *completely at random*, that is, following the (red or yellow) links of her active clusters according to no predetermined rule. In this case, at each time *t* the buyer will choose randomly one of her *k*_*j*_ first neighbors, and move toward it at time *t*+1. One of the most remarkable results of this paper is that the random strategy is far from being a losing one. In fact, it turns out that a random walk over the (active clusters of the) graph will result more effective, in terms of utility, than the analogous one influenced only by the attraction field (see Fig 5, in the extreme case Aw = 0). This unexpected result is extensively discussed later in the paper.

Finally, we describe the termination of the algorithm, that is, how a given type of consumer may end her informative journey. In this respect, we need to point out an additional feature of the selection process, which affects the dynamical rules described above. In fact, the algorithm puts a constraint on the number of times that a buyer can visit a given node of her active subgraph: this upper bound is reasonably set as equal to the degree of the node itself. We impose this limitation in accordance to the (realistic) assumption that a consumer should not travel along the same path more than once. Therefore, after *k* visits, a node with degree *k* will be “switched-off”, and it will be no longer possible to cross it.

**Fig 6:**
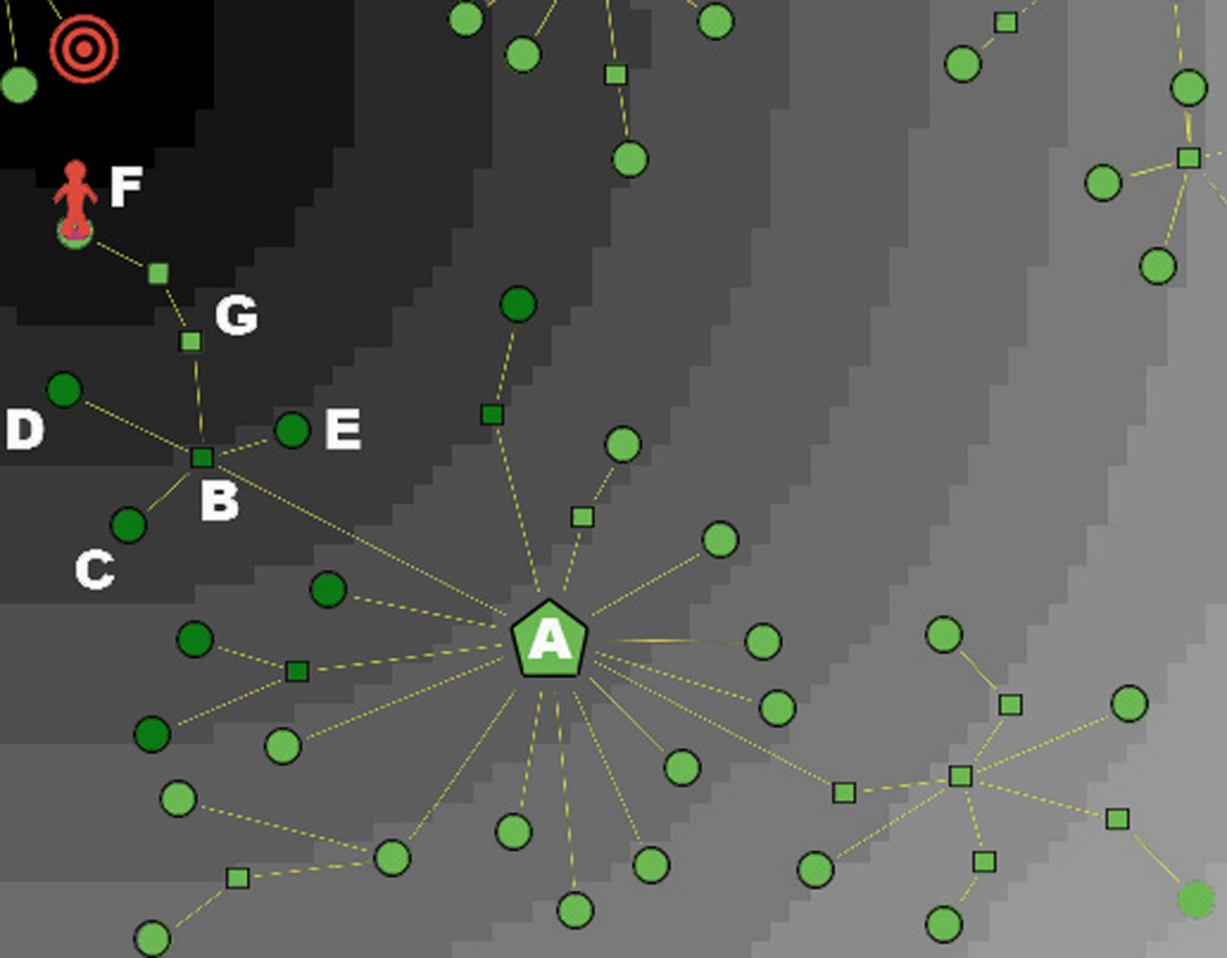
Example of a cul-de-sac. In an attempt to reach her target, the buyer is exploring a branch of her active cluster, but remains trapped in an informative cul-de-sac, and will end her journey on node *G.*

In Fig 6 we show a buyer (with awareness Aw ∼ 0.5) who, starting from the central node *A*, passes through the intermediate node *B* in order to visit the terminal nodes *C*, *D* and *E*. All visited nodes at a certain stage are colored in dark green, which stands for “switched-off”. Now imagine that, before going towards node *F*, the consumer comes back one more time to node *A* (for example, because she is moving at random). When she finally walks towards *F*, node *B* (which has degree *k* = 5) will be switched-off too, and the buyer’s fate is to remain trapped in a cul-de-sac: in fact, she will end her journey on node *G*. As a consequence of this feature of the algorithm, the consumer can terminate her journey (at time *t* = *t*_*end*_) in two different ways, described below.

(a) *The consumer remains trapped in an informative cul-de-sac*. (This situation may happen regardless of the fact that the target coincides with an existing node of the graph or not.) In this case, the consumer buys, among the products visited until the time *t*_*end*_, the product *P* corresponding to the maximum relative satisfaction, and so Sat(*P*, t*)<1. The chosen product *P* is located at the relative minimum distance *d*(*P*, P*) from the target P* (see Eq 1). Note that, since we are in the metric space $R^{2}$ with the Euclidean distance, the visited node with minimum distance from the target is essentially unique from a probabilistic point of view.
(b) *The consumer reaches her target and buys it*. This can happen only if the target coincides with an existing node that belongs to one of the active clusters. In this case, the algorithm terminates at the target P*, and the satisfaction of the consumer is given by Sat(P*, *t*_*end*_) = 1.

We end this section by defining two measures of the consumer’s performance in the whole process. The first one is the *final utility* *U*, defined as the maximum value of satisfaction obtained by the consumer at time t* ∈ [0, *t*_*end*_] during her informative journey, that is,

$$U:=\text{Sat}(P,t^{*})$$ (2)

The second one is the *total efficiency* *H* of the consumer’s experience, defined as

$$H:=\frac{t^{*}}{t_{\text{end}}}.$$ (3)

These definitions readily yield that the equality (*U*, *H*) = (1,1) only holds in one case, namely, whenever the buyer is able to reach his target (at time t* = *t*_*end*_). In all other cases, we will have 0 ≤ *U* < 1 and 0 ≤ *H* < 1, with values depending on the individual parameters Kn, *N*_Lev_ and Aw.

We aim at characterizing the ten types of consumers described in Table 1 by their respective values of *U* and *H*. Note that each simulation run of the algorithm corresponds to a single informative journey with fixed values of Kn, *N*_Lev_ and Aw. Further, the performance of each type of consumer is obviously influenced by the random initial positions of both the buyer and the target, as well as by the random selection of the active clusters. Therefore, in order to obtain statistically significant result, we need to calculate *U* and *H* for each category of buyers over many different simulation runs (events), starting from different initial conditions. The next section is devoted to a detailed description of the implementation of this procedure. Note that, although we performed simulations for many different combinations of the three main parameters, for the sake of brevity we shall only present the ten typical cases of consumers described in Table 1, since this choice does not affect the generality of our results.

## Simulation Results

Here we present and discuss the obtained results. The market structure considered in the simulations is the one represented in Fig 2, with seven clusters embedded in a satisfaction space. Note that our choice of the market structure represents a prototypical general case of monopolistic competition.

For the first five types of consumers described in Table 1 (with limited knowledge Kn = 0.5), there are three active clusters (out of seven). For each type of consumer, we consider a set of 50 simulations, called *multievents*, with different random choices of the three active clusters. Note that 50 simulations suffice since we have (73)=35 possible choices of three clusters out of seven. Further, for each multievent we perform a set of 500 simulations, with different initial positions of both buyer and target, for a total of *N* = 25000 events. Finally, over the whole set of *N* events, we compute the distributions *P*(*U*) and *P*(*H*) of final utility *U* and total efficiency *H*, respectively. The mean value of the final utility distribution is called *Social Welfare*, and is denoted by *W*; its standard deviation *σ*(*W*) is reported, too. We also calculate the distribution *P*(*t*_*end*_) of stopping times, and report its mean value *T* = 〈*t*_*end*_〉 and the corresponding standard deviation *σ*(*T*). For statistical significance, a similar set of *N* = 25000 simulations is also done for consumers with complete knowledge, corresponding to types from #6 to #10 in Table 1. Indeed, since Kn = 1 implies that the seven clusters of the market are all active and reciprocally connected, there would be no need of averaging over the 50 randomly chosen different subsets of the active clusters. We repeat the experiment only for the sake of completeness.

In what follows, we discuss the results of the simulations, separating the analysis of the following two cases: (1) *target on product*, that is, the target coincides with a product existing on the market; and (2) *target off product*, that is, the target is only ideal, since there is no product on the market that perfectly corresponds to the consumer’s goal.

### Target on product

**Fig 7:**
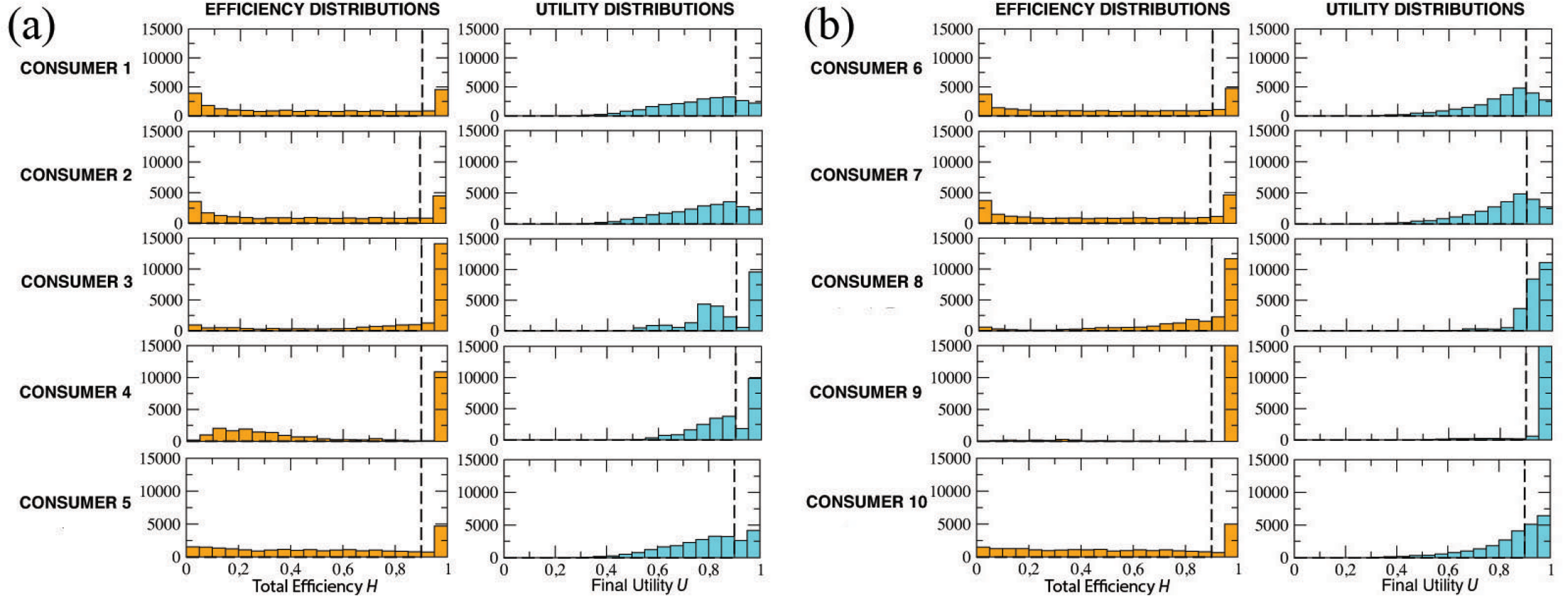
Market with “target on product”. (a) Distributions for consumers 1 to 5 (Kn = 0.5). (b) Distributions for consumers 6 to 10 (Kn = 1.0). Distributions of efficiency and final utility calculated over *N* = 25000 simulation events for the 10 types of consumers shown in Table 1. Dashed lines indicate levels of efficiency and utility higher than 0.9.

Assume that the target coincides with one of the terminal nodes of the graph. In this situation, consumers can in principle reach the target without remaining trapped in some informative cul-de-sac; however, this result strictly depends on their knowledge, awareness and discriminating ability. In Fig 7 we plot the distributions of both total efficiency and final utility for the 10 types of consumers. In particular, in panel (a) we consider consumers with limited knowledge (Kn = 0.5), whereas in panel (b) we describe consumers with complete knowledge (Kn = 1).

It is expected that group (b) performs better than group (a), with respect to both efficiency and utility. It is also far from surprising that perfectly aware buyers (Aw = 1) are in a better position than totally unaware ones (Aw = 0). In particular, only type #9—who is endowed with a perfect knowledge and awareness, and has a high discriminating ability *N*_Lev_ = 20—is able to reach her target in almost all the 25000 events, thus totaling the highest scores (equal to 1) for both *H* and *U*. Note that, however, the *y*-axis of her plots is truncated at 15000.

On the contrary, it is rather unexpected that random consumer types (#5 and #10) are in a better position than the corresponding unaware ones (#1, #2, #6 and #7), since their distributions are more shifted to the right. However, this seemingly strange effect can be rationally explained after a moment’s thought. Indeed, a consumer who moves around the graph only according to the attractiveness of various brands, with no awareness of the market and no discriminating ability whatsoever, is likely to be trapped into a “*branding loop*”: she blindly wonders around the graph, being pulled and pushed in all directions by the strength of each brand, and ends up loosing sight of her original goal. Under the same hypothesis, moving completely at random may result into a winning strategy, since independence from the attraction field will likely allow the consumer to explore quite a few nodes before stopping her informative journey.

To better appreciate the differences in performances among the various types of consumers, in Table 2 we list the following values: the percentage of events with final utility *U* > 0.9 and the percentage of events with total efficiency *H* > 0.9 (see dashed lines in all the plots of Fig 7), the social welfare *W* with its standard deviation *σ*(*W*), and the average stopping time *T* with its standard deviation *σ*(*T*).

**Table 2:** Market with “target on product”. Global quantities calculated over the *N* = 25000 simulation events for the 10 types of consumers described in Table 1. We report, in order, the following quantities: the percentage of events with final utility *U* > 0.9, the percentage of events with total efficiency *H* > 0.9, the social welfare *W* with its standard deviation *σ*(*W*), and the average stopping time *T* with the corresponding standard deviation *σ*(*T*).

Consumer	Kn	Aw	*N*_Lev_	*H*(%)>0.9	*U*(%)>0.9	*W*	*σ*(*W*)	*T*	*σ*(*T*)
#1	0.5	0	4	21	19	0.76	0.16	46	24
#2	0.5	0	20	21	20	0.76	0.15	46	24
#3	0.5	1	4	61	41	0.86	0.13	25	18
#4	0.5	1	20	44	47	0.88	0.11	21	18
#5	0.5	*random*	−	22	27	0.79	0.15	43	24
#6	1	0	4	23	27	0.80	0.14	75	42
#7	1	0	20	23	27	0.80	0.14	76	42
#8	1	1	4	56	78	0.93	0.07	14	14
#9	1	1	20	93	94	0.97	0.07	7	5
#10	1	*random*	−	23	46	0.85	0.14	60	39

As expected, the outcomes of both random consumers and those with no awareness do not depend on the number *N*_Lev_ of indifference levels, apart from insignificant statistical fluctuations. Table 2 also confirms a better performance of the group with total knowledge (Kn = 1) over that with partial knowledge (Kn = 0.5). Within these groups, it is apparent the superiority of totally aware consumers (Aw = 1) and, in particular, of consumer #9, not only concerning *H*, *U* and *W*, but also regarding the average simulation time *T*: indeed, consumer #9 reaches the target almost always (*W* = 0.97) and, in average, very quickly (*T* = 7), thus confirming the effectiveness of our algorithm in conditions of perfect knowledge. Finally, data confirms the better score of random consumers (#5 and #10) with respect to the completely unaware ones (e.g., #2 and #7), in particular for what concerns final utility.

**Fig 8:**
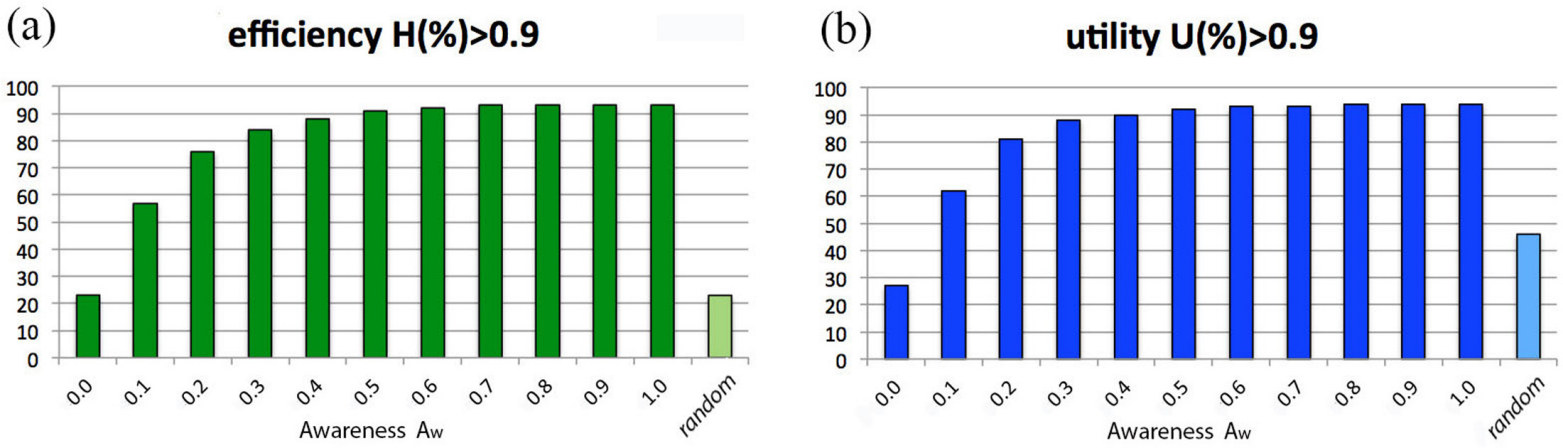
Market with “target on product”. (a) Percentage of events with total efficiency *H*(%) > 0.9. (b) Percentage of events with final utility *U*(%)>0.9. Behavior of *H*(%)>0.9 and *U*(%)>0.9 as a function of Aw, for Kn = 1 and *N*_Lev_ = 20. The case of a random consumer is also considered.

Fig 8 describes the behavior of efficiency *H* and utility *U* as a function of the consumer’s awareness Aw. Specifically, the outcomes of *H*(%)>0.9 and *U*(%)>0.9 are reported for several values of awareness, fixed Kn = 1 and *N*_Lev_ = 20; for the sake of comparison, results for random consumers are also listed. Note that scores increase along with Aw and rapidly saturate, reaching already their maximum value at Aw = 0.5. The interpretation of these results is rather natural. On one hand, there is no need to be perfectly informed consumers in order to get the maximum satisfaction from a purchase: in fact, a medium amount of information suffices. On the other hand, for scarcely informed consumers, a small quantity of information about the market is enough to overcome the performance of a random buyer. However, should one also take into account the cost needed to gather information, it is likely that a random search strategy would gain a better position in the total ranking.

We conclude the analysis of the case “target on product” by performing two important tests. The first test aims at verifying the influence of the attraction field for the various types of consumers. In Table 3 we list the percentage of events (over the total *N* = 25000 events) in which each type of consumer ends her journey within one of the seven clusters of the graph, ranked by the decreasing values of their mass *M* (these results are very robust, since the error is less than 1%).

**Table 3:** Test for attractive field in a market with “target on product”. The percentage of stops within each of the seven clusters of the graph is reported as a function of their mass *M*.

Consumer	Kn	Aw	*N*_Lev_	*M* = 33	*M* = 26	*M* = 24	*M* = 19	*M* = 17	*M* = 12	*M* = 8
#1	0.5	0	4	26	21	18	14	13	6	3
#2	0.5	0	20	29	18	17	16	11	6	3
#3	0.5	1	4	17	26	15	11	9	10	13
#4	0.5	1	20	17	23	10	11	11	11	16
#5	0.5	*random*	−	19	22	22	13	10	9	5
#6	1	0	4	32	23	20	10	9	4	2
#7	1	0	20	32	23	21	10	9	4	2
#8	1	1	4	17	20	17	16	12	11	8
#9	1	1	20	22	20	17	13	11	9	7
#10	1	*random*	−	21	22	21	12	11	8	5

As expected (see Section 2.4), only consumers characterized by Aw = 0 are visibly attracted by clusters with high masses, especially in case they are free of moving all over the graph (as it happens for types #6 and #7, who possess total knowledge): the percentage of stops is particularly high for the cluster with *M* = 33, and then it decreases along with decreasing masses. For all the other types of consumers, including random ones, the different number of stops in the various clusters is only due to obvious statistical effects: in fact, clusters with a higher mass also possess a larger number of nodes, hence the probability that a buyer stops within them is proportionally higher, even with no attraction effect whatsoever.

**Fig 9:**
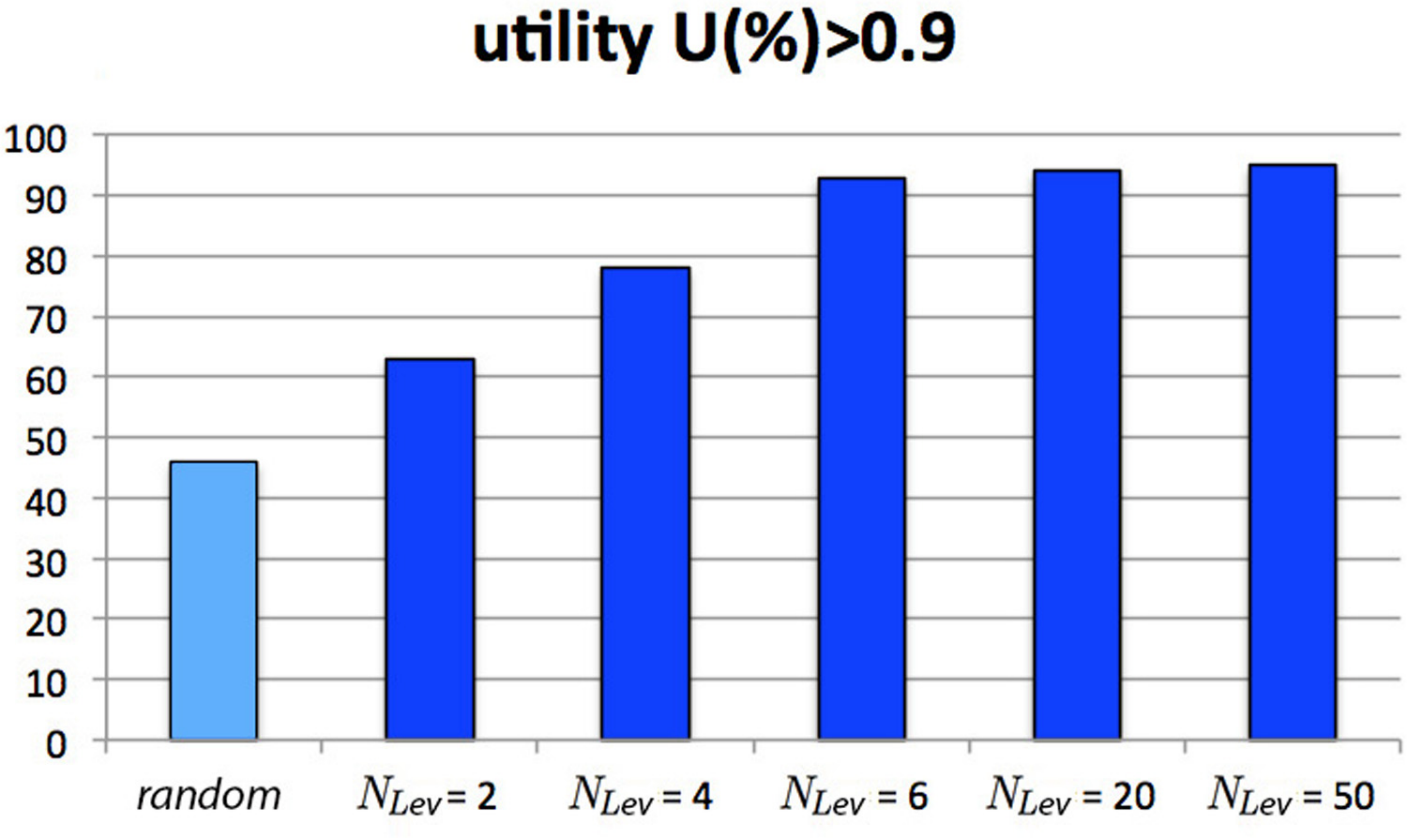
Test for the role of indifference levels in a market with “target on product”. The percentage of events with final utility greater than 0.9 is reported as a function of the number of indifference levels.

A second test concerns the effect of the number *N*_Lev_ of indifference levels, the parameter determined on the basis of the consumer’s preference structure. In Fig 9 we report the percentage of events (over the total *N* = 25000) in which perfectly informed consumers (type #9, with Kn = 1 and Aw = 1) reach a final utility greater than 0.9 as a function of *N*_Lev_. The analogous result for the random consumer (the same as in Fig 8(b)) is also reported for comparison. It is apparent, on one hand, that the presence of only two indifference levels (*N*_Lev_ = 2) does not help the informed consumer to perform much better than the random one (only 17% more). On the other hand, more than twenty indifference levels (*N*_Lev_ > 20) do not appreciably improve her performance. This explains why our simulations only take into account the cases with *N*_Lev_ = 4 and *N*_Lev_ = 20. The previous tests are quite robust, and stay substantially unchanged when the target does not coincide with an existing product. Therefore we will not repeat them in the next section.

### Target off product

Here we summarize the results of simulations—similar to those of the previous section—in the case that the target is on a point of the two-dimensional metric space which does not coincide with any terminal node of the graph. In this situation, even perfectly aware consumers cannot reach the target, and their informative journey is destined to end in a cul-de-sac (at time *t*_*end*_). However, since nothing prevents that consumers can approach the target quite early in their journey, we expect high values of final utility for consumers with Aw = 1 and Kn = 1. On the other hand, we also expect that, in the same conditions, efficiency is not so high as in the target-on-product case: in fact, for the target-off-product case, the journey goes on even after the consumer has reached her minimum distance from the target at time t*, and so the ratio t*/*t*_*end*_ might be quite lower than 1.

**Fig 10:**
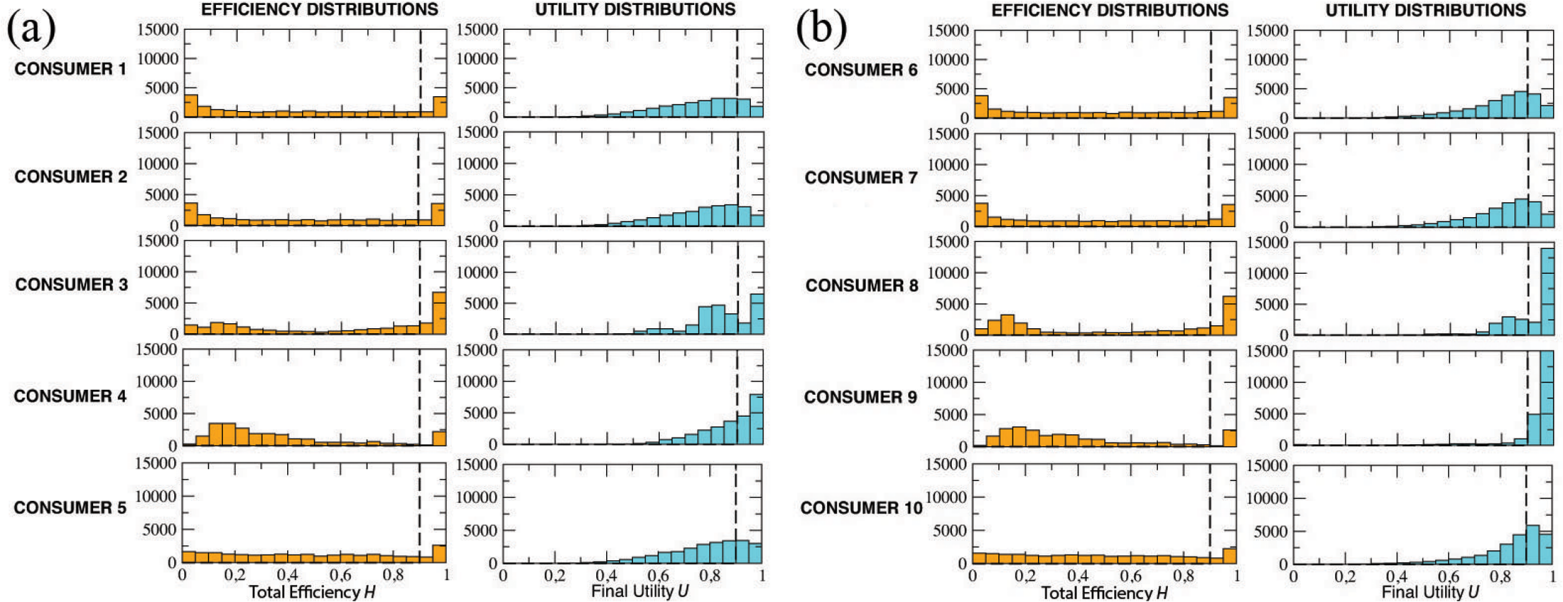
Market with “target off product”. (a) Distributions for consumer types #1-5 (Kn = 0.5). (b) Distributions for consumer types #6-10 (Kn = 1). Distributions of efficiency and final utility calculated over *N* = 25000 simulation events for the 10 types of consumers. Dashed lines indicate levels of efficiency and utility higher than 0.9.

The distributions of both the efficiency and the final utility for the 10 types of consumers is shown in Fig 10, where, as in Fig 7, in panel (a) we consider consumers with limited knowledge (Kn = 0.5), and in panel (b) we display consumers with complete knowledge (Kn = 1). The obtained results essentially confirms our expectations: group (b) performs better than group (a), in particular for what concerns the final utility; further, perfectly aware buyers (with Aw = 1) perform better than totally unaware ones (Aw = 0). It is worth noting, once again, the surprising effect of randomness, namely, the good performance of random buyers with respect to consumers with no awareness.

These trends find further support in Table 4, where we report details about the percentage of events with total efficiency *H* > 0.9 and final utility *U* > 0.9, along with social welfare and average stopping time (and the corresponding standard deviations). In general—comparing the results to those of Tables 2 and 4 shows lower scores for all observed variables (except the stopping times, which are higher) and all consumer types, thus indicating a global performance that is (naturally) worse than the analogous one in the target-on-product case.

**Table 4:** Market with “target off product”. Global quantities are calculated over *N* = 25000 simulation events for the 10 types of consumers. We report, in order, the same quantities as in Table 2: the results are very similar.

Consumer	Kn	Aw	*N*_Lev_	*H*(%)>0.9	*U*(%)>0.9	*W*	*σ*(*W*)	*T*	*σ*(*T*)
#1	0.5	0	4	18	19	0.75	0.16	49	25
#2	0.5	0	20	18	19	0.76	0.15	48	24
#3	0.5	1	4	34	33	0.84	0.12	35	15
#4	0.5	1	20	9	50	0.87	0.12	31	17
#5	0.5	*random*	−	14	26	0.78	0.15	46	25
#6	1	0	4	19	25	0.80	0.14	79	42
#7	1	0	20	19	25	0.80	0.14	79	42
#8	1	1	4	31	64	0.91	0.11	47	19
#9	1	1	20	11	89	0.94	0.10	33	19
#10	1	*random*	−	12	42	0.84	0.14	65	40

In particular, comparing the performances of consumer #9, which is naturally the best type in both the tables, one notice that in Table 4 the efficiency *H* > 0.9 drops from 93% down to 11%, while the utility *U* > 0.9 does not reach 90% (against a 94% of Table 2); at the same time, the social welfare passes from 0.97 to 0.94, and the average stopping time increases from 7 to 33 time steps. On the other hand, comparing the random consumer #10 in the two tables, one can see that the collapse of performance is less dramatic: this means that the random strategy confirms its superiority with respect to the completely uninformed one (visible by comparing consumers #10 and #7 in Table 4), and seems also to be unaffected by the position of the target (on or off product).

**Fig 11:**
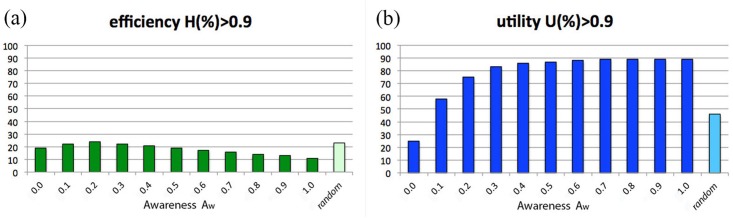
Market with “target off product”. (a) Percentage of events with total efficiency *H*(%)>0.9. (b) Percentage of events with final utility *U*(%)>0.9. Behavior of *H*(%)>0.9 and *U*(%)>0.9 as a function of Aw, for consumers with Kn = 1 and *N*_Lev_ = 20. The case of a random consumer is also considered.

In Fig 11, the behavior of *H*(%)>0.9 and *U*(%)>0.9 (for buyers with Kn = 1 and *N*_Lev_ = 20) is reported as a function of consumer awareness, as already done in Fig 8. Compared with the latter, it immediately appears that the final utility trend—shown in Fig 11(b)—remains almost unchanged (it rapidly saturates to a maximum value that is just slightly lower than in the target-on-product case), but the shape of the efficiency behavior looks now very different: indeed, due to the impossibility to reach the target, the efficiency score never exceeds 30%, staying also below the one of the random consumer for any value of awareness. Thus, considering both efficiency and utility, the random consumer’s performance results again quite effective, not only with respect to the totally unaware consumers, but also with respect those with Aw > 0: again, this is particularly relevant in view of the fact that gathering information has a cost, and so randomness appears even more convenient than what one can derive from the results of Fig 11(b).

**Fig 12:**
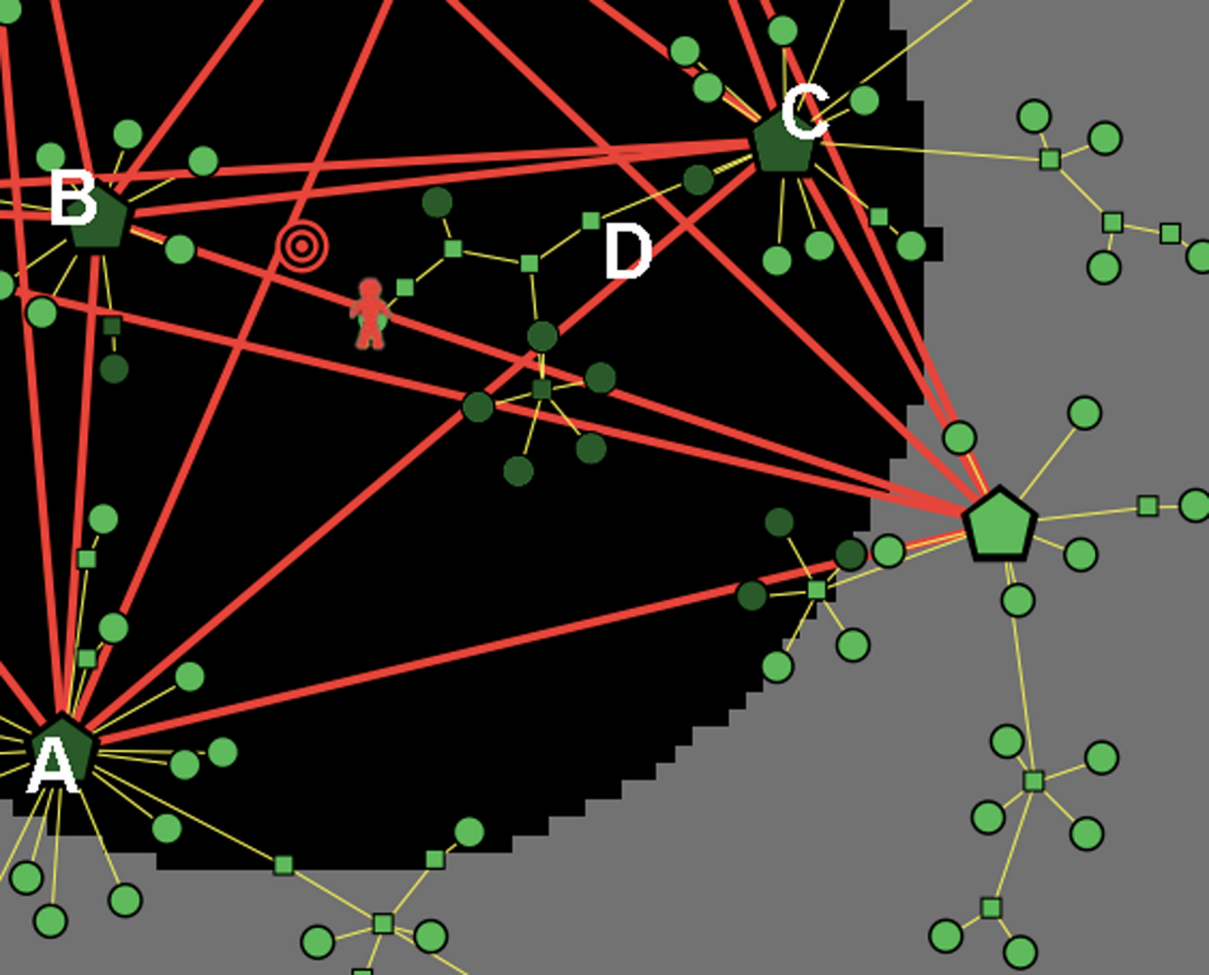
Market with “target off product”: an example of the counterintuitive effect due to a small number of indifference levels. In an attempt to reach her ideal target, a well aware and informed consumer with a low discriminating ability (type #8 of Table 4) remains trapped in a cul-de-sac, yet her efficiency stays quite high.

Finally, let us to focus the attention on a strange effect visible in both Tables 2 and 4. Some reader may have noticed that, when consumers with Aw = 1 cannot reach the target (because either Kn < 1 or the target is off product), the percentage of events with efficiency *H* greater than 0.9 seems to *decrease* when the discriminating ability of consumers increases (from *N*_Lev_ = 4 to *N*_Lev_ = 20), whereas (as expected) the final utility *U*(%)>0.9 increases. This happens, in particular, for consumer types #3 and #4 in Table 2, and types #3, #4, #8 and #9 in Table 4. Although counterintuitive at a first sight, such a behavior has a natural explanation if one deeper analyzes how the efficiency of a consumer is affected by the presence of a small number of indifference levels around the target (which is a symptom of a low discriminating ability). In Fig 12 we show an example of informative journey for a consumer with Kn = 1, Aw = 1 and *N*_Lev_ = 4, in the target-off-product case (that is, we are considering consumer #8 of Table 4). Due to her low discriminating ability, the buyer considers all the nodes within the first indifference level (colored in black) as equivalent: therefore, guided only by the degree of nodes, she wanders among the three hubs *A*, *B* and *C*, with only occasional raids down some branches of the respective clusters, until she finally ends up in the branch where is depicted in the figure. As one can see, she has reached (at, say, time t*) the product placed at the minimum possible distance from the target, and so her satisfaction—and, in turn, her utility—jumps to its (relative) maximum value (see Eqs (1) and (2)). However, her journey is bound to end soon: in fact, due to the high number of previous visits, hub *C* has been switched-off, hence the consumer’s fate is to remain trapped in a cul-de-sac. In particular, she will stop, after four more steps, on node *D* at time *t*_*end*_ = t*+4, thus producing an efficiency score very close to 1 (see Eq (3)); on the other hand, her utility score may be quite lower than 1. Translated into reality, this effect captures the behavior of an undecided (and possibly well informed) consumer who, due to her scarce discriminating ability, oscillates for a while among different brands, and suddenly decides to buy a certain product, even if it does not perfectly matches her target.

## Conclusive Remarks

In this paper we have presented a graph-based model of consumer choice, which describes the hypothetical cognitive journey that each individual experiences in the process of buying a product. The role of the causes that influence the decision is measured by means of behavioral differentiation in several parametric simulations. Our results explicitly show the relevance of information and knowledge, in the form of individual awareness, discriminating ability, and perception of market structure.

We have focused our attention on some prototypical categories of consumers, taking in account their subjectively distorted visions of the market, as it seems to happen in everyday consumption experiences. Many of our results confirm what one would naturally expect: for example, a perfect knowledge of the market structure paired with a high discrimination ability and a good individual awareness usually determines a very satisfactory choice. On the other hand, a few results of our simulations look rather surprising—and maybe intriguing—in terms of individual satisfaction, efficient strategies, and decision procedures, and therefore call for a deeper analysis of their explanation and consequences.

First of all, our model shows that consumers provided with a minimal level of knowledge and information may unexpectedly reach very high levels of utility. This appears to be in sharp contrast with the classical paradigm that “perfect information is mandatory to obtain optimal results”. However, considered that actual markets are far from being characterized by perfect information, our results provide some justification for more realistic approaches, which do not rely on perfect information as an unquestionable tenet of optimality.

Second, the results of our simulations consistently suggest that whenever consumers fail to have a minimal level of knowledge and information, random decisions will make them better off. This translates into a new type of behavioral strategy, which may operate as a sort of protective shield for the unaware category of consumers. Said differently, a consumer, who wants to avoid that social-economic forces—advertising, bandwagon effects, persuasive market power—may well defeat market attraction by employing a random approach, especially since the latter is free of charge. It is worth noting that a random behavioral model could be a source of inspiration for alternative strategies of both firms and policy makers, for example concerning new anti-trust and competition laws.

A further consideration arising from the results of our simulations concerns the emerging category of the “informed-but-undecided” consumer. In fact, for this peculiar type of buyers, it turns out that the higher their discrimination ability is, the worse their efficiency in consumption becomes. This seeming counterintuitive effect has however an explanation: with a great capacity to distinguish differentiated characteristics of goods, the final efficiency can be very high just in case of few indifference levels.

As we pointed out in the introduction, our analysis is restricted to a prototypical network configuration of the market, namely, a monopolistically competitive setting. Our choice is motivated by the fact that monopolistic competition, along with oligopoly, is one of the most recurrent structures in real markets. It would be interesting to test the robustness of our conclusions by considering also additional market configurations, such as oligopoly, perfect competition, monopoly, etc. We are also aware that an empirical validation of the model, by means of real datasets, would give further support to the above conclusions. Forthcoming research is being devoted to address these issues.
